# Polysomnographic features associated with clonazepam and melatonin treatment in isolated REM sleep behavior disorder: Time for new therapeutic approaches?

**DOI:** 10.1111/cns.14569

**Published:** 2024-02-07

**Authors:** Maria P. Mogavero, Raffaele Ferri, Sara Marelli, Giuseppe Lanza, Michele Terzaghi, Alessandra Castelnuovo, Lourdes M. DelRosso, Carlos H. Schenck, Luigi Ferini‐Strambi

**Affiliations:** ^1^ Vita‐Salute San Raffaele University Milan Italy; ^2^ Sleep Disorders Center, Division of Neuroscience San Raffaele Scientific Institute Milan Italy; ^3^ Sleep Research Centre and Clinical Neurophysiology Research Unit Oasi Research Institute – IRCCS Troina Italy; ^4^ Department of Surgery and Medical‐Surgical Specialties University of Catania Catania Italy; ^5^ Department of Brain and Behavioral Sciences University of Pavia Pavia Italy; ^6^ Unit of Sleep Medicine and Epilepsy IRCCS Mondino Foundation Pavia Italy; ^7^ University of California San Francisco Fresno California USA; ^8^ Minnesota Regional Sleep Disorders Center, Department of Psychiatry, Hennepin County Medical Center University of Minnesota Medical School Minneapolis Minnesota USA

**Keywords:** clonazepam, drugs, melatonin, polysomnographic parameters, REM sleep behavior disorder

## Abstract

**Aims:**

Although clonazepam (CLO) and melatonin (MLT) are the most frequently used treatments for REM sleep behavior disorder, the polysomnographic features associated with their use are little known. The aim of this study was to evaluate polysomnographic and clinical parameters of patients with idiopathic/isolated REM sleep behavior disorder (iRBD) treated chronically with CLO, sustained‐release MLT, alone or in combination, and in a group of drug‐free iRBD patients.

**Methods:**

A total of 96 patients were enrolled: 43 drug‐free, 21 with CLO (0.5–2 mg), 20 with sustained‐release MLT (1–4 mg), and 12 taking a combination of them (same doses). Clinical variables and polysomnography were collected.

**Results:**

Although clinical improvement was reported in all groups, MLT impacted sleep architecture more than the other treatments, with significant and large increase in N3 stage, moderate reduction in N2 and REM sleep, and moderate increase in REM latency. CLO moderately increased the percentage of both REM sleep and especially N2, while reducing N1 and wakefulness. Patients treated with both CLO and MLT did not show major changes in sleep architecture.

**Conclusion:**

These results suggest that the administration of MLT or CLO impacts (positively) on sleep parameters of iRBD patients. However, there is a need to better stratify patients, in order to treat them in a targeted manner, depending on the patient's individual sleep architecture and expected differential effects of these agents.

## Graphical abstract

In idiopathic REM sleep behavior disorder, treatment with clonazepam (CLO) was associated with decreased wakefulness and stage N1, while treatment with melatonin (MLT) was associated to decreased sleep stages N2 and REM, as well as increased sleep stage N3. Overall, the impact of MLT on sleep architecture was more evident than CLO.

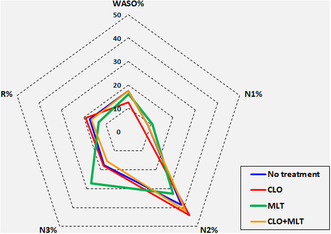

## 1 INTRODUCTION

REM sleep behavior disorder (RBD) is a parasomnia characterized by the loss of physiological muscle atonia during REM sleep (REM sleep without atonia—RSWA), resulting in the enactment of dream content, with vocalizations and complex motor behaviors,^1^ not attributable to other medical or iatrogenic conditions. It is a very complex condition, sometimes associated with other sleep disorders (such as narcolepsy), or with neurological or psychiatric disorders and use of some drugs (antidepressants, β‐blockers).^2^ RBD is very often an early sign of a neurodegenerative process, especially α‐synucleinopathy, such as Parkinson's disease, Lewy body dementia and Multiple System Atrophy, disorders for which RBD is included among their diagnostic criteria.^3^, ^4^

The diagnosis of the disorder, therefore, requires polysomnography (PSG) for the evaluation of RSWA, a required neurophysiological sign of RBD, characterized by persistent muscle tone during REM sleep (resulting in excessive tonic activity), or intermittent excessive activity in REM sleep (phasic), or both^5^; RSWA can be assessed by visual or automatic quantification methods, such as the REM sleep atonia index (RAI).^6^, ^7^

From a neurophysiological point of view, the current evidence indicates that muscle atonia during REM sleep seems to be due both to the inhibition and to the reduced activation of motor neurons (inhibited by GABA and glycinergic neurons, located in the ventromedial medulla and probably by spinal interneurons), and to the reduction or loss of excitability of motor neurons of the sublaterodorsal tegmental nucleus or subcoeruleus nucleus (due to reduction of glutamatergic, noradrenergic, dopaminergic and hypocretinergic activity).^3^, ^6^, ^8^, ^9^

Beside the effects of a dysfunction within the above neurophysiological pathways leading to RSWA, the behaviors observed in patients are often violent and the dream content is unpleasant and fearful; therefore, the complex dysfunctional network underlying the onset of RBD episodes might also involve the cortical limbic system. Physiologically, cortical activation during REM sleep is limited to a few limbic regions, including the medial entorhinal cortex, anterior cingulate cortex and dentate gyrus and the activation of these structures could produce dream scenarios. Furthermore, their activation could excite glutamatergic pyramidal neurons in the motor cortex, which in turn, in RBD are able to excite spinal motor neurons because of the dysfunctional and noneffective blockade of these signals at the brainstem level.^3^

In addition to the well‐known brainstem‐related mechanisms, an unbalanced motor cortex excitability to transcranial magnetic stimulation may be part of the RBD pathophysiology, as recently reported,^10^ also when RBD occurs in the context of an overt parkinsonian syndrome.^11^ Overall, these findings are in line with the proposed model of the retrograde influence of the motor cortex on brainstem nuclei and support the view of RBD as a widespread network dysfunction that goes far beyond the brainstem and acetylcholine alone.^8^, ^12^

To date, however, no treatment is available that is able to target specifically the above mechanism. Both nonpharmacological (such as alarm devices at the patient's bed and protection for the patient and the bed partner), and pharmacological treatment, mainly melatonin (MLT) and clonazepam (CLO),^2^, ^3^ are used off‐label because neither drug is approved by the United States Food and Drug Administration or the European Medicines Agency for RBD.^3^ The mechanism underlying the efficacy of CLO for the treatment of the behavioral episodes of RBD is not clear; its main adverse events (dose dependent) are: somnolence, enuresis, gait disturbances, cognitive alterations and dizziness^3^, ^13^; similarly, the mechanisms of action of MLT on behavioral episodes of RBD are unknown, but this agent seems to be better tolerated than CLO, as it can rarely induce headache and somnolence.^3^

A recent review showed that 66.7% of 1026 patients with RBD reported improvements with CLO and 32.9% of 137 patients with RBD reported improvements with MLT; moreover, the authors pointed out that in reality the effects of these treatments could sometimes be overestimated, as attributable to a placebo effect.^14^ In fact, several experts agree on the need to conduct further pharmacological trials, in which the objective measurement of PSG data in RBD should be the primary outcome, rather than the use of assessment scales or subjective diaries,^15^ stratifying patients into different subtypes of RBD,^16^ allowing the use of targeted therapies.

Finally, the recently drafted guidelines of the American Academy of Sleep Medicine for the management in the clinical practice of RBD indicate the administration of CLO, immediate‐release MLT, and pramipexole (the latter in case of a high periodic leg movements during sleep [PLMS] index), all able to induce clinical improvement, but only MLT and pramipexole also on the frequency of the disorder, concluding that further research is needed to better understand the effect of these therapies.^17^

Based on this clinical evidence and literature and the scarcity of studies conducted on the direct comparison of the effects of CLO and MLT used for the treatment of RBD on PSG and clinical parameters, we planned this study with the aim to evaluate PSG and clinical parameters of patients with idiopathic/isolated RBD (iRBD) treated with CLO, sustained‐release MLT, or both in combination, and in a control group of drug‐free iRBD patients, to better understand the objective differential sleep parameters associated with their use and what may be the best therapeutic indications for each of these compounds.

## 2 SUBJECTS AND METHODS

### 2.1 Subjects

A total of 96 iRBD patients (82 males and 14 females, age range 50.9–83.2 years) were enrolled in this study: 43 drug‐free, 21 patients taking at bedtime chronically (>1 month) CLO (0.5–2 mg), 20 patients taking MLT sustained‐release alone at bedtime (1–4 mg), and 12 taking a combination of CLO and MLT sustained‐release (same doses as above, at bedtime). Table 1 shows the demographics of the patient groups. A careful diagnosis of iRBD was made, in all patients, following the current international criteria.^1^ None of the patients was affected by moderate or severe sleep apnea and none was taking any other pharmacological treatment that could impact hypnic architecture. Clinical variables collected were: age, age at onset, disease duration, treatment duration, clinical global impression scale (severity, or CGI‐S, and improvement, or CGI‐I), and mini‐mental state examination.^18^ This study was approved by the local ethics committee and conducted according to the World Medical Association Declaration of Helsinki; all subjects provided their informed consent.

**TABLE 1:** Demographic features of patients.

	Males			Females		
	*N*	Mean	SD	*N*	Mean	SD
No treatment	40	68.8	5.99	3	71.3	6.16
CLO	15	69.7	5.55	6	63.5	9.30
Melatonin	18	69.1	7.55	2	68.8	15.04
CLO + melatonin	9	66.0	8.32	3	68.1	10.37

### 2.2 Video PSG

Video PSG (vPSG) was recorded following the American Academy of Sleep Medicine criteria^5^ and included electroencephalogram (at least one frontal, one central, and one occipital channel, referred to the contralateral mastoid); electrooculogram, electromyogram (EMG) of the submentalis muscle, EMG of the right and left tibialis anterior muscles, respiratory signals, a single lead electrocardiogram, and video and audio recording. Epochs and all sleep parameters were scored by a certified sleep technologist or board certified sleep physician, according to standard criteria.^5^

For the computer quantitative analysis of the submentalis muscle EMG activity we used an established automatic scoring algorithm to compute the above‐mentioned RAI.^19^, ^20^ Mathematically, RAI can vary from 0 (the complete absence of EMG atonia), to 1 (stable EMG atonia). RAI correlates significantly with the percentage of epochs of REM sleep without atonia detected by the method by Lapierre and Montplaisir^21^, ^22^ and performs comparably to other visual methods to quantify RSWA.^7^, ^22^

In order to classify the severity of RBD episodes, we evaluated motor behavior events during REM sleep on vPSG recordings and graded them visually and polysomnographically on an event‐to‐event basis, by means of the REM sleep behavior disorder severity scale (RBDSS).^23^ According to this scale, the location of movements was categorized as follows: “0” = no visible movement; “1” = slight movements or jerks “2” = movements involving proximal extremities, including violent behavior; “3” = axial involvement including bed falls. Vocalizations were rated as “1” for present or “0” for absent. The final RBDSS score was determined by the highest score obtained in each vPSG recording. In order to treat statistically these results, we slightly modified the final score by adding to the movement location category (0–3) the value of 0 in the absence of vocalizations or 0.5 in their presence; in this way, we obtained an 8‐level grading for RBDSS (0–3.5).

### 2.3 Statistical analyses

Continuous variables obtained in the four groups of patients were compared by means of the analysis of variance (ANOVA) or the analysis of covariance (ANCOVA), as appropriate, followed by post‐hoc comparison of individual group pairs by means of the Tukey honestly significant difference test. This study was based on a convenience sample for which reason, a reliable preliminary sample size analysis was not possible. Moreover, in order to attenuate the possible effects of the multiple comparisons carried out and avoid the negative effect of the conservative correction methods, also the effect size *f* was computed for each comparison; with this method, and following Cohen's indications,^24^ an *f* = 0.1 denotes a small effects size, *f* = 0.25 indicates a medium effect size, and *f* ≥ 0.4 characterizes a large effect size. The comparison of categorical variables obtained from the same groups of subjects was done by means of the Fisher exact test. The significance level was set at *p* < 0.05.

## 3 RESULTS

The sex composition of the groups (Table 1) was not statistically different at the Fisher test, as well as age (Table 2). Among the clinical variables considered in this study, listed in Table 2, only the treatment duration (significantly shorter in the MLT group than in the others) and CGI‐S showed significant differences between the groups considered (moderate‐to‐large effect size), with patients taking MLT showing a lower degree of clinical severity (≈3 = mildly ill) than those drug‐free or taking CLO (≈4 = moderately ill). However, the CGI‐I was reported to be “much improved” or “minimally improved” in all treated patients, without statistically significant differences between the groups. Age at onset of iRBD, disease duration, RBD severity scale score, and Mini‐Mental Examination score were not significantly different between the four groups.

**TABLE 2:** Comparison of clinical parameters found in the four groups of subjects.

	No treatment (Free, *n* = 43)		Clonazepam (CLO, *n* = 21)		Melatonin (MLT, *n* = 20)		Clonazepam + melatonin (CLO + MLT, *n* = 12)		ANOVA	Effect size	Tukey HSD
	Mean	SD	Mean	SD	Mean	SD	Mean	SD	*p*	*f*	Post‐hoc
Age, years	69.0	5.96	67.9	7.16	69.1	7.93	66.5	8.41	<0.690	0.178	
Age at onset, years	63.6	8.38	61.5	8.66	64.2	6.05	59.6	6.33	<0.300	0.205	
Disease duration, years	4.4	3.38	6.2	4.12	4.7	4.51	6.6	4.38	<0.221	0.217	
Treatment duration, years	—	—	2.3	1.56	0.4	0.46	2.6	3.08	<0.0015	0.752	MLT < CLO = CLO + MLT
RBD severity scale	1.5	1.08	1.2	1.18	1.6	0.83	1.3	0.91	<0.696	0.147	
Clinical Global Impression scale, Severity	3.9	0.52	3.9	0.70	2.9	1.95	3.8	0.75	<0.003	0.354	MLT < Free = CLO
Clinical Global Impression scale, improvement	—	—	2.7	0.77	2.4	0.75	2.5	0.67	<0.352	0.183	
Mini‐mental state examination	27.9	1.32	27.3	2.18	28.1	1.85	27.4	2.46	<0.578	0.154	

Table 3 shows the results of the comparison of vPSG parameters obtained in the four groups of patients. In this case ANCOVA was run with the CGI‐S score as a covariate (treatment duration was not used because not applicable in the drug‐free group), in order to correct for the possible effect of this parameter on the results. This was needed for the other clinical variables which were not significantly different between the groups. Several parameters were found to be accompanied by a statistically significant difference (confirmed by a corresponding moderate to a large effect size). REM latency was longer, and number of stage shifts less, in patients taking MLT than those drug free; sleep stage N1 was shorter and N2 longer in patients taking CLO than in those taking MLT, the latter had also a lower percentage of this stage than drug‐free patients and those taking CLO + MLT. Patients taking MLT also showed a higher amount of sleep stage N3 (both in terms of minutes and percentages) than that of all the other groups, while the group of patients taking CLO + MLT in combination showed significantly less PLMS than those taking MLT or drug free. Also, the comparison of REM sleep quantity showed a tendentially significant trend to be slightly more represented in patients taking CLO and less in those taking MLT; however, the post‐hoc comparisons did not reveal any significant differences in the comparisons between each pair of groups.

**TABLE 3:** Comparison of vPSG parameters found in the four groups of subjects.

	No treatment (Free, *n* = 43)		Clonazepam (CLO, *n* = 21)		Melatonin (MLT, *n* = 20)		Clonazepam + Melatonin (CLO + MLT, *n* = 12)		ANCOVA	Effect size	Tukey HSD
	Mean	SD	Mean	SD	Mean	SD	Mean	SD	*p*	*f*	post‐hoc
Time in bed, min	443.0	25.01	454.5	18.90	453.4	33.91	457.6	27.72	<0.252	0.222	
Sleep period time, min	408.6	48.52	424.0	29.86	424.5	40.54	419.1	42.27	<0.579	0.181	
Total sleep time, min	338.0	63.68	371.5	45.37	358.5	80.40	348.9	93.74	<0.189	0.184	
Sleep latency, min	27.7	42.19	19.9	12.96	21.8	20.71	32.1	30.31	<0.884	0.145	
Rem latency, min	86.3	44.23	89.2	52.66	132.9	94.12	86.7	72.78	<0.016	0.271	MLT > Free
Stage shifts/h	16.3	6.66	13.2	4.98	10.2	3.81	15.2	4.34	<0.005	0.470	MLT < Free
Awakenings/h	5.7	3.99	4.2	3.04	4.3	1.83	5.1	2.95	<0.370	0.223	
Sleep efficiency, %	76.2	13.00	81.6	8.32	79.1	16.50	76.8	20.85	<0.342	0.141	
Wakefulness after sleep onset, min	70.6	37.46	52.4	29.11	66.0	55.78	70.2	73.43	<0.245	0.139	
Sleep stage N1, min	36.5	19.06	26.9	14.71	45.5	24.26	37.4	16.96	<0.075	0.319	CLO < MLT
Sleep stage N2, min	159.3	46.12	187.8	49.61	138.9	53.97	176.3	66.86	<0.047	0.308	CLO > MLT
Sleep stage N3, min	70.2	31.22	74.3	34.20	117.1	64.35	65.4	25.71	<0.005	0.458	MLT > Free = CLO = CLO + MLT
Sleep stage R, min	71.8	30.31	82.5	46.71	57.0	20.76	69.9	31.10	<0.046	0.250	
Wakefulness after sleep onset, %	17.6	9.95	12.5	7.31	16.2	14.03	17.4	21.05	<0.154	0.143	
Sleep stage N1, %	8.8	4.18	6.3	3.32	10.7	5.41	8.9	3.78	<0.045	0.342	CLO < MLT
Sleep stage N2, %	38.6	8.73	44.2	10.74	32.6	11.79	41.6	14.87	<0.024	0.183	MLT < Free = CLO = CLO + MLT
Sleep stage N3, %	17.6	8.33	17.9	8.79	27.2	14.01	15.6	6.54	<0.020	0.413	MLT > Free = CLO = CLO + MLT
Sleep stage R, %	17.4	6.63	19.2	9.68	13.4	4.47	16.6	7.59	<0.029	0.267	
Periodic leg movements during sleep index	33.4	33.25	20.4	29.05	37.1	32.30	6.7	8.71	<0.042	0.360	CLO + MLT < Free = MLT
REM Sleep Atonia Index	0.778	0.184	0.804	0.181	0.689	0.220	0.673	0.228	<0.084	0.249	

**FIGURE 1:**
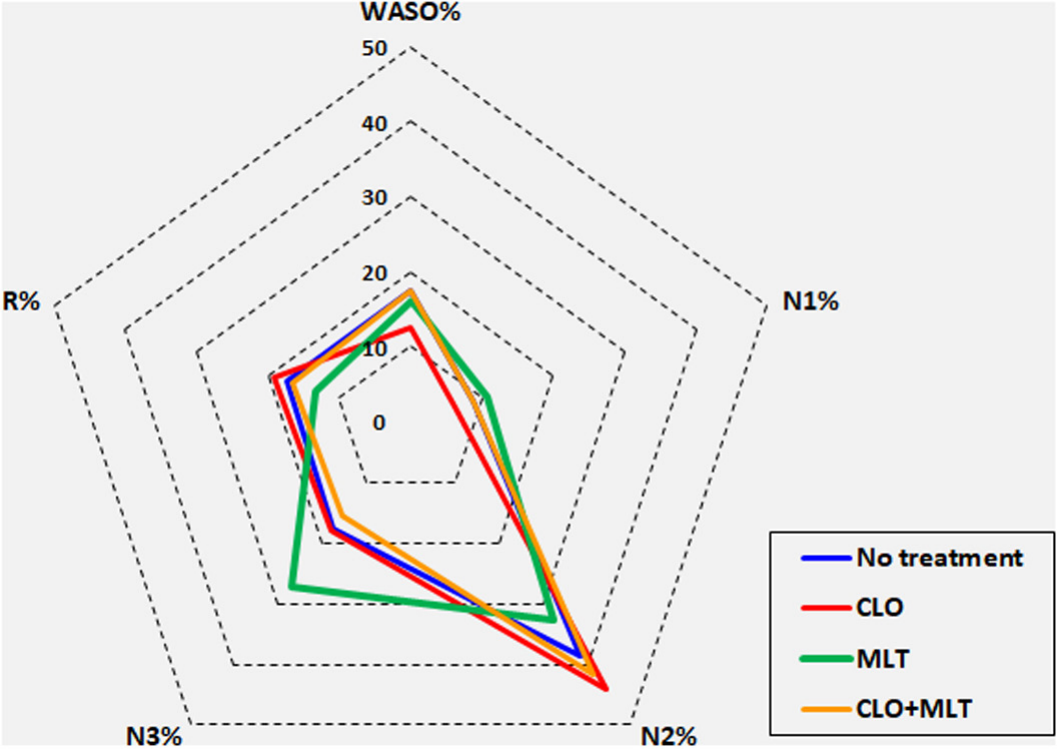
Graphic representation of sleep stage distribution in the four groups of patients.

Figure 1, shows in a graphic way, the differences in sleep stage percentage pattern in each group, with a clear tendency to show decreased wakefulness and stage N1, associated with CLO treatment, and decreased sleep stages N2 and REM, as well as increased sleep stage N3, with MLT treatment. Overall, it can be seen that the impact of MLT on sleep architecture seems to be somewhat more evident than that of CLO, beside the different sleep stage change pattern.

Finally, all groups showed low average values of RAI, as expected, and there were no statistically significant differences between drug‐free patients or taking CLO, MLT, or a combination of them. However, this comparison was characterized by a moderate‐to‐large effect size, mainly driven by the higher RAI values in the group taking CLO; this was also confirmed by the fact that 40% of patients taking CLO had RAI ≥0.9 (within the normal range) while only 16.6% of the remaining groups, pooled together, showed RAI ≥0.9 (Fisher's exact test *p* = 0.019).

## 4 DISCUSSION

The aim of this study was to assess the sleep architecture of patients with iRBD treated with sustained‐release MLT, CLO and sustained‐released MLT in association with CLO, comparing them with each other and to drug‐free iRBD patients.

In fact, in clinical practice, the most widely used treatments are CLO (at a dosage of 0.25–2 mg) and MLT (at a dosage of 3–12 mg),^17^, ^25^, ^26^ although they act on the disorder through a mechanism not yet well understood. Although there is a need to better know the rationale for the use of these drugs, the studies conducted in the literature on their effect on the PSG parameters of iRBD are very few, sometimes on small series^27^ and only one very recent work has evaluated 16 patients treated with sustained‐release MLT versus 18 patients treated with CLO, finding significant changes in sleep structure only for CLO (with increased N2 stage and reduced of N3 and REM) and a visually assessed reduction in RSWA.^28^

On the other hand, a meta‐analysis evaluated the effects of the different treatments administered to RBD patients on PSG parameters, including a total of only 13 studies, of which two with CLO, four with MLT, three with Ramelteon, three with pramipexole and one with rotigotine (among these, the study with the largest series enrolled 39 patients).^27^ CLO increased the percentage of N2 sleep, while MLT was associated with significant improvements in sleep efficiency and reduction in both phasic and tonic muscle activity; RSWA seemed to improve significantly with the use of ramelteon (MLT agonist); dopamine agonists demonstrated, as expected, improvements of PLMS, but not of other PSG parameters.^27^

Therefore, there is no doubt about the importance of carrying out further investigations on this topic,^29^ aimed however at understanding how these drugs can act on the pathophysiology of iRBD.

### 4.1 Melatonin

Although the American Academy of Sleep Medicine has made a “conditional” recommendation to use immediate‐release MLT for the treatment of iRBD in adults,^17^ it has recently been reported that also sustained‐release MLT can be effective in iRBD, at a dosage of 2 mg,^28^ similar to the average dosage used in the current study.

In our study, the CGI‐S showed significant differences between the groups considered, showing that patients taking MLT had a lower degree of clinical severity than drug‐free iRBD patients or those taking CLO, although RBDSS in the MLT group was higher. In this respect, it should be noted that CGI‐S and RBDSS assess different features; while CGI‐S is based essentially on the anamnesis and clinical assessment of patients, RBDSS is based on the evaluation of RBD episodes occurring during a single vPSG recording. The results obtained at the RBDSS are also taken into consideration, as well as all other available info, when the CGI‐S or CGI‐I are determined.

We found that MLT, above all, determines an impact on sleep architecture, compared to both controls and to the other treated groups (Table 3, Figure 1), with a significant increase in N3 sleep stage, reduction of N2 and REM sleep and increase in REM latency, while it does not determine any change in RSWA.

Only a few studies in the literature have evaluated the effect of MLT in iRBD, agreeing that it improves sleep efficiency and sometimes symptoms and muscle tone during REM (depending on the dosage used and duration of treatment)^27^, ^30^, ^31^; it has also been reported that it seems that MLT might restore muscle atonia, suggesting that it may act at a more basic level of the disorder than CLO.^32^ In fact some authors have hypothesized that it could directly potentiate GABAA receptor tonic transmission at motor neurons to decrease muscle tone.^33^ However, we could not confirm this in our case series.

Importantly, in the regulation mechanism of sleep homeostasis, the main markers of the circadian rhythm are internal body temperature and MLT, while the main markers of homeostatic sleep pressure are nonrapid eye movement sleep (NREM) and slow‐wave sleep (SWS)^34^, ^35^, ^36^; furthermore, the rostromedial tegmental nucleus (which projects to the locus subcoeruleus) is essential for NREM sleep and homeostatic regulation and exerts an important inhibitory control on the mesencephalic dopaminergic system, likely contributing to the regulation of sleep–wake behavior.^37^

On the other hand, in RBD, a disorder connected to neurodegenerative pathologies such as Parkinson's disease, Lewy body dementia and multiple system atrophy,^3^, ^4^ a degeneration of the nucleus subcoeruleus^38^ has been highlighted as an important structure needed to preserve the physiological muscle atonia during REM sleep,^3^, ^6^ as well as in modulating SWS and participating to homeostatic processes.^37^ In addition, a recent study has shown that iRBD could be associated with an alteration in the expression of the *Per2* and *BMAL1 CLOCK* genes, with delayed MLT secretion and implication of circadian rhythms in the pathogenesis of the disorder^39^; on the other hand, the *CLOCK/BMAL1* complex also regulates cerebral redox homeostasis, which could be one of the mechanisms linking the alteration of circadian clock to neurodegeneration.^40^

In this context, it is interesting to mention the study by Kunz et al.,^30^ who suggested that the chronic (≥6 months) use of 2 mg sustained‐release MLT with a chronotype‐corrected chronobiotic protocol (always‐at‐the‐same‐clock time, 10–11 p.m.) might induce an improvement of RBD symptoms lasting for years, even decades. These findings certainly need independent replication but also indicate the opportunity to further investigate on the effectiveness of MLT and on the best treatment protocol with this agent.

Past studies of sleep architecture in iRBD have shown a significant increase in the percentage of SWS, whereby the authors hypothesized a dysregulation of the central nervous system, rather than an adaptive energy conservation mechanism associated with SWS.^41^

The administration of MLT could therefore act as a modulator of the circadian process (possibly altered in iRBD), in an attempt to act on sleep homeostasis through the regulation of SWS and NREM sleep; this mechanism could be a possible explanation for the findings associated with MLT found in our study, in which we observed a significant increase in sleep stage N3, with a consequent reduction in sleep stages N2 and REM and an increase in REM latency. Moreover, the reduction in the percentage of REM sleep, likely with consequent reduction of dream activity, could be the reason for the clinical improvement observed in these patients in our study as well as others in the literature^27^ and documented by the CGI‐I; while the dramatic increase in SWS could explain why patients treated with MLT reported an improvement in sleep quality not only compared to controls but also compared to iRBD treated with other CLO or CLO + MLT.

Finally, it must be acknowledged that MLT treatment was significantly shorter than the other two treatments in this study, because of its observational nature; although it lasted on average for 4.8 months, being thus definitely chronic, we cannot exclude that might have influenced the results obtained, at least to some extent.

### 4.2 Clonazepam

Patients treated with CLO showed a greater representation of REM sleep and especially of sleep stage N2, with reduction of N1 and wakefulness after sleep onset, compared to controls, while sleep stage N3 was similar to that of drug‐free patients. CGI‐I showed clinical improvement, although slightly, but not significantly, less than that reported in patients treated with MLT; again, no changes in RAI were found.

Clonazepam is used in clinical practice for reducing the frequency of disturbing dreams with violent and frightening content, vocalizations and vigorous movements during REM sleep,^42^ although some authors have shown that such a treatment does not seem to have effect on dream content,^43^ as it was hypothesized in other studies.^44^ On the other hand, to date only few studies have been conducted on the effect of CLO on PSG parameters in patients with iRBD,^27^, ^44^ which have shown a significant decrease in the instability of the N1 and N2 sleep stages, especially in long‐term treatment,^45^ but have not shown drug‐induced variations on RSWA,^19^, ^22^, ^46^ also suggesting that the therapeutic effect of CLO is likely to act on supratentorial rather than subtentorial networks, reducing the negative effects of brainstem dysfunction on supratentorial regions, without affecting the pathogenetic core of the disease.^46^

Current evidence indicates that CLO might decrease phasic muscle activations without restoring muscle atonia, suggesting its action on glutamatergic neurons of the motor cortex or their relays in pontine and medullary reticular formation and spinal cord,^33^ which always seem to be implicated in the pathogenesis of a network disorder^11^, ^33^ and in agreement with previous assumptions on the effect of CLO on supratentorial networks.^46^

As known, benzodiazepines (category to which CLO belongs) cause, under physiological conditions, an increase in sleep stage N2 and a reduction in SWS and REM sleep.^47^ According to this, in patients with iRBD treated with CLO, we observed an increase in N2; however, there was no change in SWS and, paradoxically, an increase in the percentage of REM sleep was observed.

Naturally, these results must be interpreted considering what is known about the pathogenesis of iRBD and the alterations found in the sleep architecture of these patients, which seems to show an increase in the percentage of SWS^41^, ^45^; our findings indicating no changes in SWS in patients treated with CLO could therefore be attributable to the fact that this treatment reduces SWS, which is generally higher than normal in these patients.

Any propensity of CLO for inducing rapid tolerance in certain patient groups does not apply to the treatment of RBD and NREM parasomnias, in which rapid tolerance to CLO has not been demonstrated. For example, in a study of 136 adult patients (*n* = 52 with RBD; *n* = 69 with NREM parasomnias) who received CLO nightly for a mean 3.5 (± 2.4) years, there was no statistically significant difference in initial versus the final mean dose: 0.77 mg (± 0.46) versus 1.10 mg (± 0.96).^48^

Also, we have carefully studied the effects of CLO on sleep neurophysiology in the past,^44^, ^45^ finding effects on both vPSG and RBDSS similar to those reported in the current new study; this prompted us to hypothesize that, possibly, CLO acts on the oneiric content, perhaps making it less violent.^44^ However, we were also able to demonstrate subtle but significant changes in the EEG spectral content during REM sleep and in its instability,^46^ in addition to the changes we had already reported on NREM sleep instability.^44^

The increase in REM sleep observed in patients with CLO may be due to the fact that this treatment has different effects on the mechanisms regulating sleep homeostasis from MLT and that patients with iRBD have abnormal sleep homeostasis, with absence of the suppression of beta rhythms during REM sleep (and consequent increase in cortical excitation).^46^, ^49^ In addition, the reduction in SWS induced by CLO in patients who present with an increase in SWS when drug free might cause a rebound effect of REM sleep,^50^ as if these patients experienced sleep deprivation.

It is interesting to note, however, that RAI was only slightly changed in patients using CLO, which might be supported by the longer and, perhaps, more stable REM sleep associated with this treatment.^46^, ^50^

However, the complex dysfunctional network underlying RBD may also involve other areas, such as the cortical limbic system^3^, ^6^ and there are still important gaps in our knowledge about the initiation and maintenance of REM sleep and the transition from NREM to REM sleep^51^, ^52^; thus, further studies are needed to better understand the observed findings.

### 4.3 MLT in combination with CLO

Patients treated with CLO + MLT did not show major changes in sleep architecture compared to controls, but this could be expected, based on what we have described above about the changes associated with MLT or CLO alone, somewhat opposite to each other: the former increases N3 and decreases N2 and REM sleep; the second has no effect on sleep stage N3 and is associated with an increase in N2 and REM sleep. The clinical improvement observed with the association of these two agents is therefore attributable to the aforementioned mechanisms of the individual treatments, without in fact impacting on the sleep architecture.

However, in these patients the best response for reducing PLMS was observed, compared to the other groups; this is very interesting considering that, in 1996, Schenck et al.^53^ reported that iRBD patients who phenoconverted to a parkinsonian disorder had significantly much higher PLMS index at baseline than iRBD patients who remained iRBD at follow‐up. In addition, the presence of PLMS has also been correlated with an increase in the percentage of RSWA in REM sleep, suggesting a greater severity of the disorder in these patients^54^ and that PLMS during sleep increase with age^55^, ^56^ and are associated with striatal neurodegeneration and dopamine deficiency.^57^

The fact that the association of CLO and MLT acts positively on PLMS is a proof that they may be due to a complex mechanism not known, which however could involve supra and subtentorial networks.^58^

### 4.4 Limitations

Limitations of this study were related to its observational nature and involved the relatively wide range of treatment dosage, and disease duration and severity. The treatment duration was significantly shorter in the MLT group and we could not adequately control for an eventual effect of this difference; however, the average duration of 4.8 months seems to be a period long enough to consider the treatment with MLT as chronic, as in the other two treatments.

Although all patients had a vPSG performed when the diagnosis was made, because of the sometimes very long disease duration, this initial vPSG was not available in all of them for various reasons, mainly technical. This did not allow us to carry out a vPSG comparison between baseline and treatment conditions in the same subjects; this analysis would have been of interest, but we also believe that a better planned prospective study is needed in the future. In a previous observational study on a small group of patients taking CLO, for whom baseline and treatment vPSG recordings were available (with different time lags between them), we already reported vPSG and clinical effects that were in line with those found in this new study.^44^

Moreover, the sample size could not be established prior to the execution of the study; however, we ran a post‐hoc power analysis using the observed effect sizes for the significant comparisons found (Tables 2 and 3), which ranged from moderate to large values, giving a range of statistical power from 49.9% to 97.7%, for alpha 0.05, with our total sample size of 96. In particular, most comparisons were associated to effect sizes *f* ≈ 0.3–0.35, corresponding to a statistical power of 67.1%–81.4%. Thus, the post‐hoc power analysis confirmed that our statistical analysis had an acceptable power, excellent in some cases with *f* > 0.4 (minutes and percentage of sleep stage N3, and REM sleep latency).

On the other hand, the observational nature of this study can also be viewed as a strength because it analyzes the changes in PSG parameters associated with RBD treatment in the real clinical practice. In addition, our total sample size of iRBD patients is also a factor consolidating the conclusions that can be drawn from our findings.

As already reported by a taskforce of the International RBD Study Group, assessment of vPSG holds promise but is costly and needs further elaboration.^2^ For this reason, controlled trials with vPSG are scarce in this field and cross‐sectional studies like ours are critically needed in order to pave the way to controlled trials and provide initial data on which the future controlled study can establish primary and secondary neurophysiological outcomes.

## 5 CONCLUSIONS

In conclusion, our study is the first to focus on the changes associated with the use of MLT and CLO, in monotherapy or in combination, in sleep architecture in patients with iRBD (not focusing solely on RSWA), with the aim to better understand how these therapies can have a therapeutic effect on the disorder. The results of our study suggest, on one hand, that there is a rationale for their administration in this pathology, based on its known pathogenetic mechanisms and, on the other hand, the need to better stratify patients, to administer these therapies in a targeted manner, depending on the characteristics of the patient sleep architecture, in the context of precision medicine.

Moreover, in consideration of a wide variability of scenarios and clinical evolution in iRBD (also in the context of neurodegenerative processes), the knowledge of the possible effects of these treatments on the different sleep stages and their administration depending on the patients sleep architecture, could also represent a valid aid in the modulation of the sleep structure for the purpose of a possible prevention and/or mitigation of the clinical effects of the underlying neurodegenerative processes. Finally, in light of these findings and of the considerations from this discussion, another very important aspect concerns the evaluation of the timing of MLT administration (for example, by evaluating the dim light MLT onset) in iRBD patients, in order to optimize the therapeutic outcome, as also discussed by Kunz et al.^30^ However, further studies are needed to confirm these hypotheses.

## FUNDING INFORMATION

This study was partially supported by a fund from the Italian Ministry of Health “Ricerca Corrente” RC n. 2764043 (to R.F.).

## CONFLICT OF INTEREST STATEMENT

The authors declare no conflicts of interest.

## PATIENT CONSENT STATEMENT

Written informed consent was obtained from the study participants.

## Data Availability

Anonymized data of this study will be shared upon reasonable request from a qualified investigator.
